# Combination of c-reactive protein and squamous cell carcinoma antigen in predicting postoperative prognosis for patients with squamous cell carcinoma of the esophagus

**DOI:** 10.18632/oncotarget.18667

**Published:** 2017-06-27

**Authors:** Ji-Feng Feng, Sheng Chen, Xun Yang

**Affiliations:** ^1^ Department of Thoracic Surgery, Zhejiang Cancer Hospital, Hangzhou 310022, China; ^2^ Key Laboratory Diagnosis and Treatment Technology on Thoracic Oncology, Hangzhou 310022, China

**Keywords:** esophageal squamous cell carcinoma (ESCC), c-reactive protein (CRP), squamous cell carcinoma antigen (SCC), cancer-specific survival (CSS), prognosis

## Abstract

**Background:**

We initially proposed a useful and novel prognostic model, named CCS [Combination of c-reactive protein (CRP) and squamous cell carcinoma antigen (SCC)], for predicting the postoperative survival in patients with esophageal squamous cell carcinoma (ESCC).

**Methods:**

Two hundred and fifty-two patients with resectable ESCC were included in this retrospective study. A logistic regression was performed and yielded a logistic equation. The CCS was calculated by the combined CRP and SCC. The optimal cut-off value for CCS was evaluated by X-tile program. Univariate and multivariate analyses were used to evaluate the predictive factors. In addition, a novel nomogram model was also performed to predict the prognosis for patients with ESCC.

**Results:**

In the current study, CCS was calculated as CRP+6.33 SCC according to the logistic equation. The optimal cut-off value was 15.8 for CCS according to the X-tile program. Kaplan-Meier analyses demonstrated that high CCS group had a significantly poor 5-year cancer-specific survival (CSS) than low CCS group (10.3% vs. 47.3%, *P* <0.001). According to multivariate analyses, CCS (*P* =0.004), but not CRP (*P* =0.466) or SCC (*P* =0.926), was an independent prognostic factor. A nomogram could be more accuracy for CSS (Harrell's c-index: 0.70).

**Conclusion:**

The CCS is a usefull and independent predictive factor in patients with ESCC.

## INTRODUCTION

Esophageal cancer (EC) is one of the most fatal types of cancer, leading to over 406,800 deaths worldwide and more than 200,000 deaths in China annually [1, 2]. The histological types are different between China and western countries [3]. Esophageal squamous cell carcinoma (ESCC) is the predominant pathological type in China, which covers more than 90% of all EC cases [3, 4]. Radical esophagectomy remains the treatment of choice for localized disease, however, the prognosis is still poor [5].

It has increasingly been recognized that inflammation plays a critical role in cancer [6, 7]. Serum c-reactive protein (CRP) is a sensitive biomarker for inflammation. Recent studies revealed that CRP was associated with prognosis in several cancers [8–10]. However, the prognostic role of CRP in EC is still controversial [11–14]. As we know, CRP is influenced by various non-cancer related conditions. In addition, anti-inflammatory medicines and/or other medications may also potentially affect the level of serum CRP.

Serum tumor markers play a key role in cancer diagnosis and prognosis. Therefore, in order to improve the survival time for cancer patients, it is essential to explore relevant tumor markers in various cancers. Squamous cell carcinoma antigen (SCC) is a tumor marker for squamous cell carcinoma [15]. Recently, SCC is also widely used in a variety of cancers, such as cervical cancer and head and neck cancer [16, 17]. Nevertheless, to date, few data regarding SCC in patients with EC are available, and its role remainscontroversial [18–20].

As we know, both CRP and SCC may influenced by various non-cancer related conditions, and combination of CRP and SCC could therefore minimise the potential basis. Therefore, we initially proposed a useful and novel prognostic model, named CCS (Combination of CRP and SCC), for predicting the prognosis for patients with ESCC. To the best of our knowledge, no study so far has evaluated the prognostric value of CCS in other cancers as well as ESCC, which makes our study the first of its kind. In addition, we attempt to establish a predictive nomogram to predict the survival prediction in patients with ESCC.

## RESULTS

**Figure 1:**
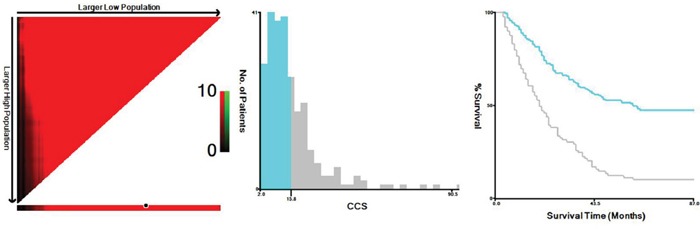
X-tile analysis for CCS The optimal cut-off point highlighted by the black circle in the left panels is shown on a histogram of the entire cohort (middle panels, 15.8), and a Kaplan-Meier plot (right panels).

Of the total number of patients, 37 (14.7%) were women and 215 (85.3%) were men. The mean age was 58.5 ± 8.2 years (range 36-80). The logistic regression equation was defined as follow: Y=0.101CRP+0.639SCC-0.686. Thus, CCS=CRP+0.639/0.101SCC=CRP+6.33 SCC. The optimal cut-off value for CCS was 15.8 according to the X-tile program (Figure 1). The relationships between CCS and clinical characteristics were shown in Table 1.

**Table 1:** The relationship between CCS and clinical characteristics in ESCC patients

	Cases (n)	CCS ≤ 15.8 (n, %)	CCS > 15.8 (n, %)	P-value
Age (years)				0.097
≤ 60	151	105 (63.6)	46 (52.9)	
> 60	101	60 (36.4)	41 (47.1)	
Gender				0.158
Female	37	28 (17.0)	9 (10.3)	
Male	215	137 (83.0)	78 (89.7)	
Tumor length (cm)				0.016
≤ 5.0	188	131 (79.4)	57 (65.5)	
> 5.0	64	34 (20.6)	30 (34.5)	
Tumor location				0.297
Upper	17	11 (6.7)	6 (6.9)	
Middle	117	71 (43.0)	46 (52.9)	
Lower	118	83 (50.3)	35 (40.2)	
Vessel invasion				0.089
Negative	203	138 (83.6)	65 (74.7)	
Positive	49	27 (16.4)	22 (25.3)	
Perineural invasion				0.067
Negative	204	139 (84.2)	65 (74.7)	
Positive	48	26 (15.8)	22 (25.3)	
Differentiation				0.114
Well	37	26 (15.8)	11 (12.6)	
Moderate	156	107 (64.8)	49 (56.3)	
Poor	59	32 (19.4)	27 (31.1)	
T stage				<0.001
T1-2	94	77 (46.7)	17 (19.5)	
T3-4	158	88 (53.3)	70 (80.5)	
N stage				0.024
N0	146	104 (63.0)	42 (48.3)	
N1-3	106	61 (37.0)	45 (51.7)	
CRP (mg/L)				<0.001
≤ 10.0	180	154 (93.3)	26 (29.9)	
> 10.0	72	11 (6.7)	61 (70.1)	
SCC (ng/ml)				<0.001
≤ 1.50	179	140 (84.8)	39 (44.8)	
> 1.50	73	25 (15.2)	48 (55.2)	

ESCC=esophageal squamous cell carcinoma; CCS=combination of CRP and SCC; CRP= c-reactive protein; SCC= squamous cell carcinoma antigen

Statistical methods: The Pearson Chi squared test.

**Figure 2:**
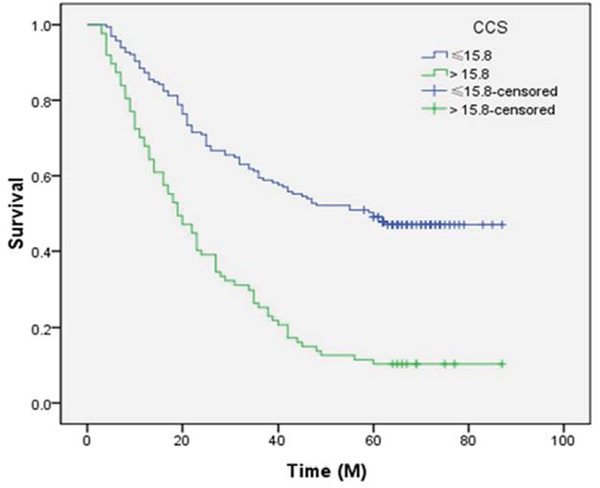
Kaplan-Meier CSS curves stratified by CCS Patients with CCS ≤15.8 had a significantly better 5-year CSS than patients with CCS >15.8 (47.3% vs. 10.3%, *P* <0.001).

Kaplan-Meier analyses demonstrated that high CCS group had a significantly poor 5-year CSS than low CCS group (10.3% vs. 47.3%, *P* <0.001) (Figure 2). Multivariate analyses revealed that CCS (*P* =0.004), but not CRP (*P* =0.466) or SCC (*P* =0.926), was an independent prognostic factor (Table 2). In addition, T stage (*P* =0.045) and N stage (*P* <0.001) were also significant independent predictors for CSS (Table 2).

**Table 2:** Univariate and multivariate analyses in ESCC patients

	Univariate analysis	*P*-value	Multivariate analysis	*P*-value
	HR (95% CI)		HR (95% CI)	
Age (years)		0.967	-	-
>60 vs. ≤60	1.007 (0.737-1.376)			
Gender		0.138	-	-
male vs. female	1.425 (0.892-2.274)			
Tumor length (cm)		0.292	-	-
>5.0 vs. ≤5.0	1.204 (0.852-1.700)			
Tumor location		0.379	-	-
lower vs. upper/middle	1.120 (0.870-1.442)			
Vessel invasion		0.002		0.742
positive vs. negative	1.752 (1.225-2.504)		1.068 (0.722-1.579)	
Perineural invasion		0.018		0.957
positive vs. negative	1.553 (1.079-2.233)		1.011 (0.682-1.499)	
Differentiation		0.060	-	-
poor vs. well/moderate	1.278 (0.989-1.650)			
T stage		<0.001		0.045
T3-4 vs. T1-2	2.193 (1.559-3.087)		1.472 (1.009-2.149)	
N stage		<0.001		<0.001
N1-3 vs. N0	2.671 (1.957-3.644)		1.975 (1.385-2.816)	
CRP (mg/L)		<0.001		0.466
>10.0 vs. ≤10.0	2.152 (1.563-2.963)		1.193 (0.743-1.914)	
SCC (ng/ml)		0.016		0.926
>1.50 vs. ≤1.50	1.492 (1.077-2.066)		0.981 (0.660-1.458)	
CCS		<0.001		0.004
>15.8 vs. ≤15.8	2.853 (2.090-3.896)		2.092 (1.272-3.441)	

ESCC=esophageal squamous cell carcinoma; CRP= c-reactive protein; SCC= squamous cell carcinoma antigen; CCS=combination of CRP and SCC; HR=hazard ratio; CI=confidence interval

Statistical methods: Univariate and multivariate analyses.

**Figure 3:**
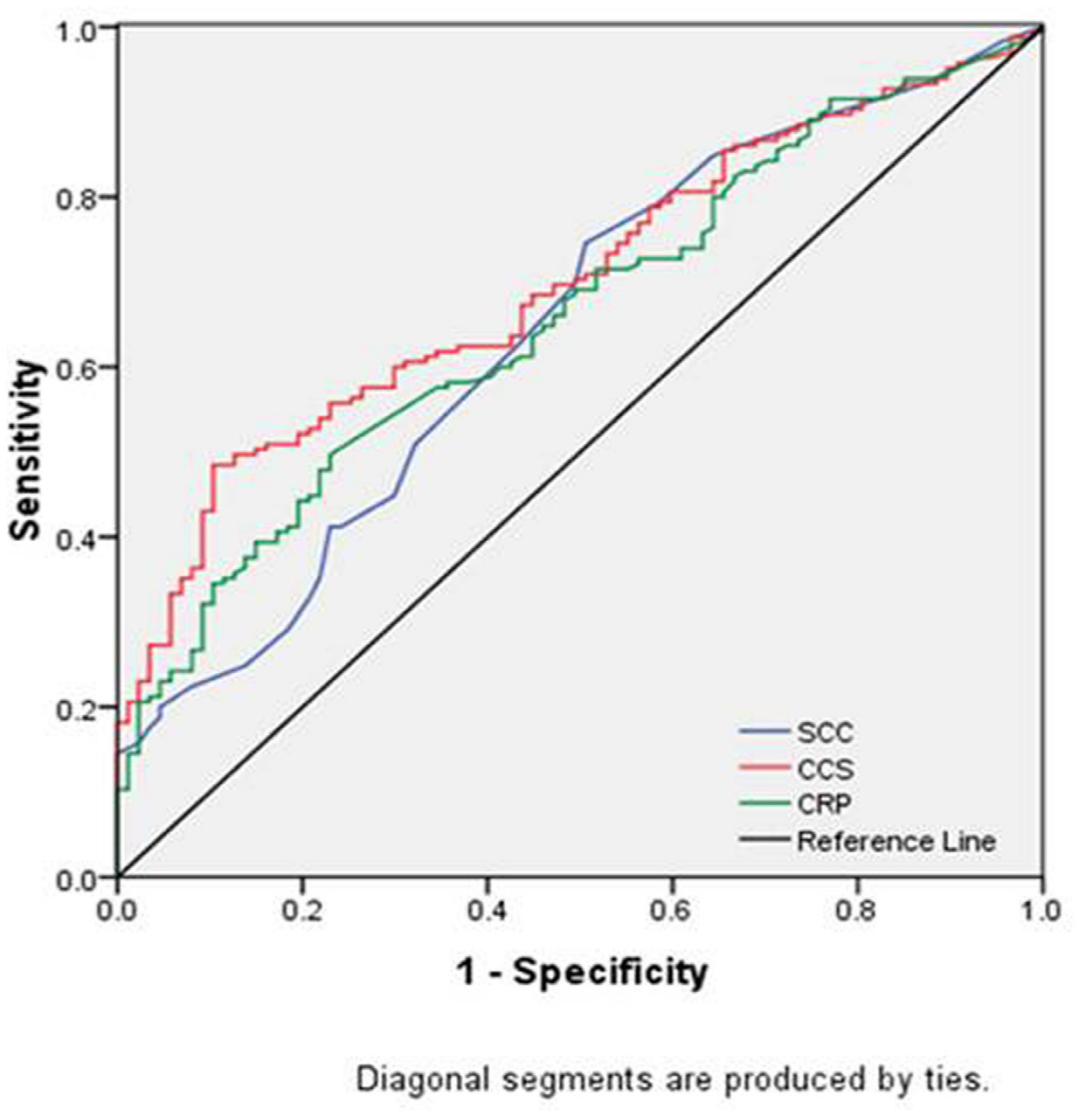
ROC curves for CSS prediction The AUC was 0.699 (95% CI: 0.635-0.763, *P* <0.001) for CCS, 0.659 (95% CI: 0.591-0.727, *P* <0.001) for CRP and 0.645 (95% CI: 0.574-0.716, *P* <0.001) for SCC, respectively.

The areas under the curve (AUC) was 0.699 (95% CI: 0.635-0.763, *P* <0.001) for CCS, 0.659 (95% CI: 0.591-0.727, *P* <0.001) for CRP and 0.645 (95% CI: 0.574-0.716, *P* <0.001) for SCC, respectively (Figure 3). Although the discrimination ability of the CCS was higher than CRP or SCC, the Z tests revealed that there were no significant differences for each other (CCS vs CRP, Z=1.428, *P*=0.153; CCS vs SCC, Z=1.912, *P*=0.056; CRP vs SCC, Z=0.278, *P*=0.781). The results demonstrated that the CCS predicts survival similar to CRP or SCC. However, multivariate analyses revealed that CCS (*P* =0.004), but not CRP (*P* =0.466) or SCC (*P* =0.926), was an independent prognostic factor.

**Figure 4:**
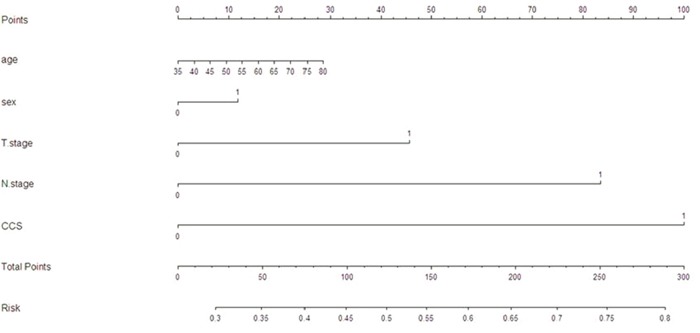
Nomogram model for death risk prediction The Harrell's c-index for CSS prediction was 0.70.

To predict the risk for patients with ESCC, a novel nomogram model was established by independent prognostic factors, including T stage, N stage and CCS, combined with age and sex (Figure 4). It can predict the probability of death for patients with ESCC. The Harrell's c-index for CSS prediction was 0.70.

## DISCUSSION

To our knowledge, this is the first study to evaluate the prognostic role of CCS (combination of CRP and SCC) in predicting prognosis for patients with resectable ESCC. In the present study, a novel prognostic model (CCS) was conducted based on CRP and SCC and was shown to be an independent predictor for patients with resectable ESCC.

Recently, Camp et al. [21] initial developed a graphical method, named X-tile plot, to illustrate a method of dividing a single cohort into training and validation subsets. They concluded that the X-tile plot can present a new tool for the assessment of biological relationships and discover cut-points based on marker expression. In the current study, the optimal cut-off value for CCS was calculated by a X-tile program, which was 15.8 (Figure 1). Then patients were divided into two groups. The results demonstrated that high CCS group had a significantly poor 5-year CSS than low CCS group (10.3% vs. 47.3%, *P* <0.001).

It has increasingly been recognized that inflammation plays a critical role in cancer. CRP is an acute phase protein with systemic inflammation. More and more studies demonstrated that serum CRP is associated with prognosis in several cancers, including EC [8–10, 13, 14]. Recently, we conducted a meta-analysis revealed that CRP were significantly correlated with survival in patients with EC [22]. In our study, patients with CRP ≤10.0 mg/L had a significantly better 5-year CSS than patients with CRP >10.0 mg/L (41.7% vs. 16.7%, *P* <0.001). However, CRP was not an independent prognostic factor in multivariate analyses (*P*=0.466). SCC is a tumor marker for squamous cell carcinoma. However, the role for SCC in patients with EC remainscontroversial [18–20]. A meta-analysis revealed that elevated SCC do not be associated with poor survival [23]. In our study, patients with SCC ≤1.5 ng/ml had a significantly better 5-year CSS than patients with SCC >1.5 ng/ml (38.0% vs. 26.0%, *P* =0.014). However, multivariate Cox regression model revealed SCC was still not an independent prognostic factor (*P*=0.926).

Recently, Chen et al. [24] demonstrated that SCC and CRP as prognostic biomarkers in recurrent oral cavity squamous cell carcinoma. They revealed that patients with the high levels of SCC and CRP had a significantly worse overall survival. This observation is in line with data from our study. In our study, however, multivariate Cox regression model revealed neither SCC nor CRP was independent prognostic factor. The other biomarker called ADAR1 has been discussed as a potential new biomarker for ESCC [25]. ADAR1, also known as RNA editase, has attracted increasing attention in recent years. They concluded that over-expressed ADAR1 correlates to shorter survival time of ESCC patients. It has been reported that interferons induce the upregulation of ADAR1 [26], therefore raising the possibility that ADAR1 serves as an antiviral defense mechanism against inflammation. However, in comparison with the ADAR1, the CCS is easy to measure routinely because of its low cost and convenience.

As we know, both CRP and SCC may influenced by various non-cancer related conditions, and combination of CRP and SCC could therefore minimise the potential basis. Therefore, we initially proposed a useful and novel prognostic model (CCS) in patients with ESCC. In our study, CCS (*P* =0.004), but not CRP (*P* =0.466) or SCC (*P* =0.926), was an independent prognostic biomarker. Additionally, we showed a better discrimination for CCS in terms of HR than CRP and SCC. From this point of view, the CCS may have additional prognostic value over the CRP or SCC with regard to predicting CSS in ESCC patients.

It is widely agreed that T stage and N stage are strong, independent prognostic factors in patients with EC. Due to the relatively small number of patients, we divided the patients into two groups regarding T stage (T1-2 vs T3-4) and N stage (N0 vs. N1-3) like previous published articles [27–29]. In our study, we also demonstrated that T stage (*P*=0.045) and N stage (*P*<0.001) were independent prognostic factors.

It is well know that nomogram could establish a simple and graphic representation of a statistical predictive model [30]. In the current study, therefore, we attempt to establish a predictive nomogram model to predict the probability that the death risk for ESCC patients based on T stage, N stage and CCS combined with age and sex. The nomogram performed well in predicting CSS by c-index (0.70).

The potential limitations of the present study include the use of a retrospective analysis and the relatively small number of patients. Furthermore, in our study, we excluded patients who had received neoadjuvant treatment, which may have influenced the result. In addition, our study revealed that CCS is an independent factor, however, it should be kept in mind that CCS itself alone without other variables may not associate with prognosis. Therefore, larger prospective studies will need to be performed to confirm these preliminary results.

In summary, the CCS is a usefull and independent predictive factor in patients with ESCC. Based on the results of our study, we believe that CCS was superior to CRP or SCC as a more precise prognostic biomarker in ESCC.

## MATERIALS AND METHODS

Between January 2005 and December 2008, a total of 252 patients with resectable ESCC were included in the current retrospective study. The eligibility criteria were included: (1) ESCC was confirmed by histopathological examination; (2) curative surgery with margins histologically free of disease; (3) without preoperative neoadjuvant treatment; (4) without any form of acute infection or chronic inflammatory disease; and (5) preoperative serum CRP and SCC were obtained before surgery within one week. The patients in the current study were staged according to the 7th edition of the American Joint Committee on Cancer Cancer Staging [31]. The study was approved by the Ethics Committees of Zhejiang Cancer Hospital.

The standard surgical approach included the Ivor Lewis and the McKeown procedure. The lymphadenectomy included two-field and three-field lymphadenectomy. In the current study, most of patients underwent two-field lymphadenectomy. Three-field lymphadenectomy was performed only if the cervical lymph nodes were thought to be abnormal. As the role of postoperative adjuvant treatment was controversial during that period, adjuvant therapy was not mandatory. The most frequent adjuvant chemotherapy included 5-fluorouracil and cisplatin and the median postoperative radiation dose was 50 Gy.

Data on preoperative laboratory examination were extracted in our medical records. The serum CRP and SCC were taken within one week prior to surgery. The cut-off value for CRP and SCC were 10 mg/L and 1.5 ng/ml according to the previous studies [13, 14, 20]. The CCS was calculated by combined CRP and SCC according to the logistic equation. In the current study, a cancer-specific survival (CSS) analysis was ascertained. The last follow-up was 30 June 2013.

### Statistical analysis

The optimal cut-off value for CCS was calculated by a X-tile program [21]. The Pearson Chi squared test was used to determine the significance of differences for dichotomous variables. Kaplan-Meier methods were used to analyse CSS. Univariate and multivariate analyses were performed to analyse the prognostic factors. A receiver operating characteristic (ROC) curve for survival prediction was plotted to verify the CSS prediction. The area under curve (AUC) was used as an estimation of diagnostic accuracy. A Z test was performed to compare the significant differences for CCS, CRP and SCC. A nomogram model was also established and the predictive accuracy was evaluated by Harrell's concordance index (c-index) [32]. Statistical analyses were conducted with SPSS 17.0 (SPSS Inc., Chicago, IL, USA) and R 3.2.3 software (Institute for Statistics and Mathematics, Vienna, Austria).
